# Supplementary material for: Ptn–Ov synergistic sites on MoOx/γ-Mo2N heterostructure for low-temperature reverse water–gas shift reaction
Source: Nat Commun. 2022 Oct 3;13:5800. doi: 10.1038/s41467-022-33308-7 (PMC9530113; doi:10.1038/s41467-022-33308-7)
Supplement: Supplementary file 1 — Supplementary Information [file 41467_2022_33308_MOESM1_ESM.pdf]

Supplementary Information for

Pt_n-O_v synergistic sites on MoO_x/γ-Mo_2N heterostructure for low-temperature reverse water–gas shift reaction

Hao-Xin Liu[1], Jin-Ying Li[1], Xuetao Qin[2], Chao Ma[3], Wei-Wei Wang[1], Kai Xu[1], Dequan Xiao[4], Ding Ma[2]*, Qiang Fu[5]*, Chun-Jiang Jia[1]*

[1]Key Laboratory for Colloid and Interface Chemistry, Key Laboratory of Special Aggregated Materials, School of Chemistry and Chemical Engineering, Shandong University, Jinan, 250100, China.

[2]College of Chemistry and Molecular Engineering, Peking University, Beijing, 100871, China

[3]College of Materials Science and Engineering, Hunan University, Changsha, 410082, China.

[4]Center for Integrative Materials Discovery, Department of Chemistry and Chemical and Biomedical Engineering, University of New Haven, West Haven, CT 06516.

[5]School of Future Technology, University of Science and Technology of China, Hefei 230026, China.

These authors contributed equally: Hao-Xin Liu, Jin-Ying Li, Xuetao Qin.

*Corresponding author: jiacj@sdu.edu.cn; qfu3@ustc.edu.cn; dma@pku.edu.cn

Table of Contents

Supplementary Methods
Supplementary Figures
Supplementary Tables
Supplementary References

Supplementary Methods:

Computational Methods

Spin-polarized density functional theory (DFT) calculations were carried out using the Vienna ab initio simulation package (VASP)[1,2]. The projector augmented-wave method[3] was employed and the exchange-correlation interactions were described by the GGA-PBE functional[4]. The energy cutoff for plane-wave basis-set expansion was set to 500 eV. A 1 × 2 × 1 Monkhorst−Pack mesh grid[5] was used to sample the Brillouin zone for structural optimization and a 3 × 6 × 1 one for calculating the density of states. The supercell contained enough vacuum layer in the vertical direction. Structural relaxations were performed until the maximum residual force on each atom was less than 0.03 eV/Å. Transition states were located by using the climbing-image nudged elastic band and the dimer methods[6,7] with a force criterion of 0.10 eV/Å. Each transition state had been confirmed via vibrational mode analysis and the corresponding imaginary frequencies were listed in Supplementary Table 4. All structures were visualized using the program VESTA[8].

The adsorption energy was defined by using the following expression:

$$E_{ads} = E_{(surf+mol)} - E_{(surf)} - E_{(mol)} \quad (1)$$

where $E_{(surf)}$ and $E_{(mol)}$ are the energies of substrates and isolated molecules, respectively, and $E_{(surf+mol)}$ represents the energy of the combined systems upon adsorption. It meant that a negative E_{ads} value corresponds to exothermic adsorption.

The differential charge density was calculated by using the following formula:

$$\Delta\rho = \rho_{(surf+mol)} - \rho_{(surf)} - \rho_{(mol)} \quad (2)$$

where $\rho_{(surf+mol)}$ is the electron density of the combined system, while $\rho_{(surf)}$ and $\rho_{(mol)}$ represent the electron densities of the substrate and the adsorbed molecule, respectively, with their geometries fixed as those in the combined system.

Simulation Model

In the theoretical calculations, the simulation model adopted in our previous work[9]

was still employed. In brief, to construct the interface between the MoO_3 (010) adsorbed layers and the γ-Mo_2N (111) substrate, a certain degree of strain was applied to the MoO_3 (010) thin layers. The main change in the lattice, concretely, is to alter the angle of 32.46° in the figure below to 30.00°, to match the lattice of the γ-Mo_2N (111) substrate. This model well reproduced the very low formation energy of oxygen vacancies in the adsorbed MoO_3 (010) layers, in consistence with the experiments[9].

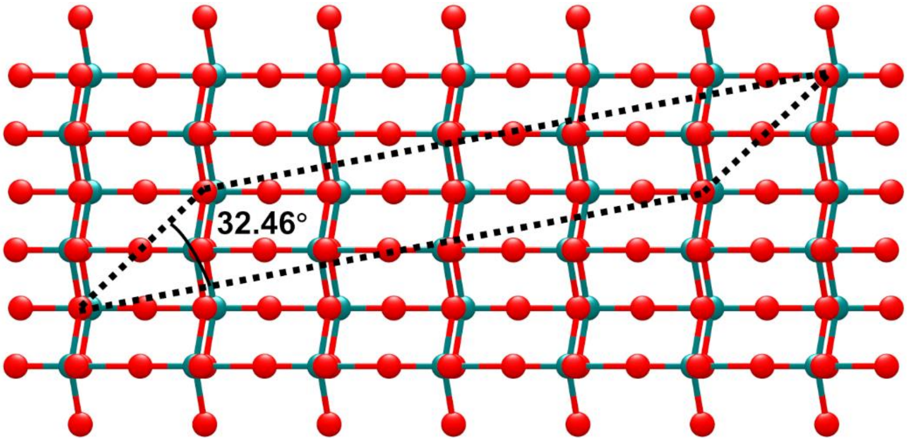

Lattice angle of the MoO_3 (010) layers.

In the original simulation model, a three-layer MoO_3 (010) and a few buffer layers of molybdenum oxide were deposited on the γ-Mo_2N (111) substrate, which contained seven atomic layers (Figure S15b in Supplementary Ref. 9). A Pt_4 cluster, which was used to represent the Pt nanoparticles in the experiments, was deposited on the MoO_x adsorbed layers after the removal of one outermost oxygen atom, and in this way, a catalytic interfacial system was constructed. Compared with the simulation model in Supplementary Ref. 9, in this work, only one atomic layer, instead of the original seven atomic layers, was included in the bottom Mo_2N part, to decrease the computational cost in the reaction pathway exploration. Since our calculations had shown that removing a few bottom Mo_2N (111) layers did not change the adsorption energies of adsorbates on Pt_4/MoO_x, such a simplification of the computational model will not affect any conclusion obtained by the simulations.

Supplementary Figures:

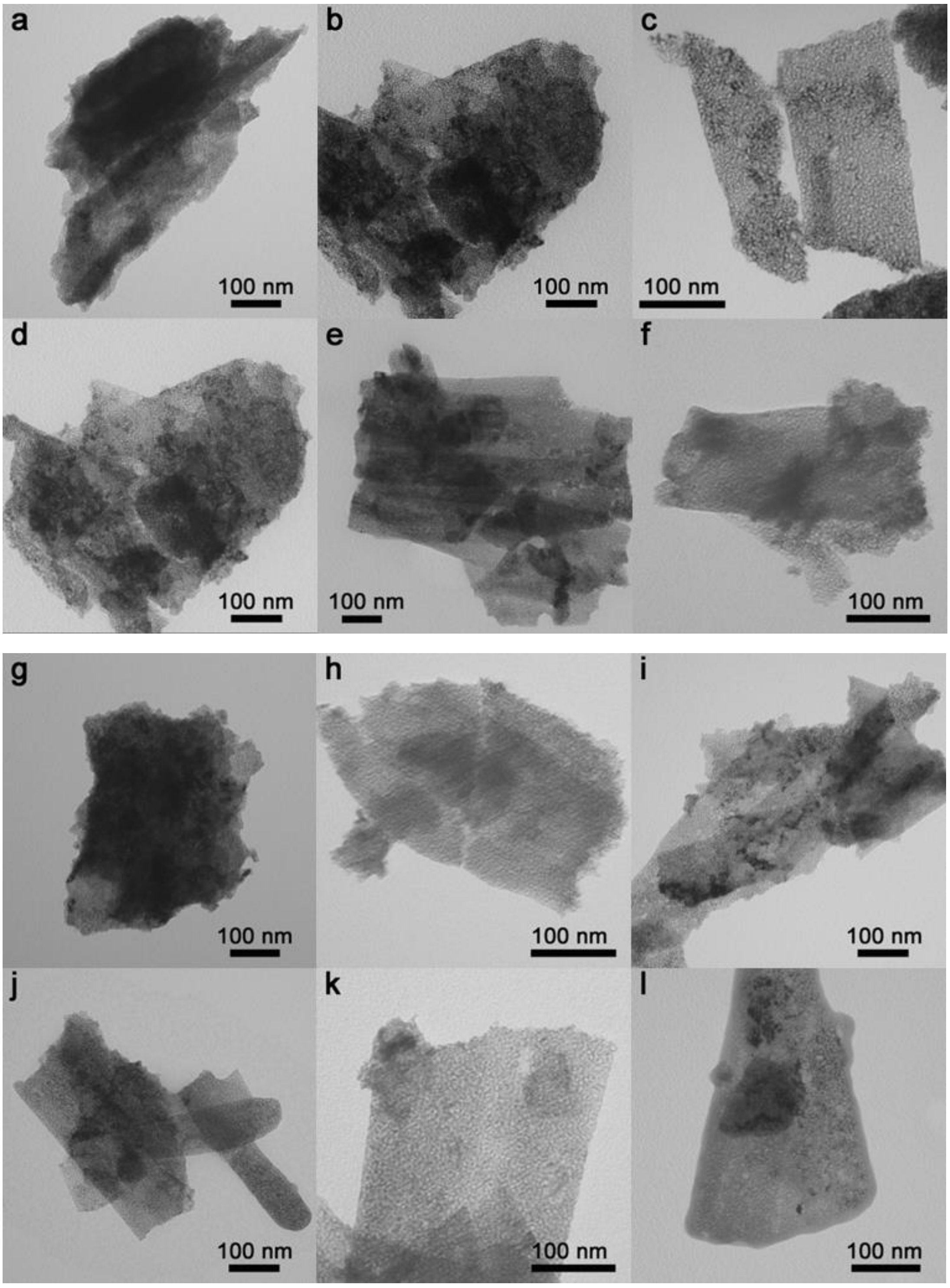

Supplementary Figure 1. TEM pictures of all Pt-MoO_3/Mo_2N catalysts. (a, g) fresh and used pure Mo_2N supports; (b, h) fresh and used 0.2Pt-MoO_3/Mo_2N catalysts; (c, i) fresh and used 0.3Pt-MoO_3-Mo_2N catalysts; (d, j) fresh and used 0.5Pt-MoO_3/Mo_2N catalysts; (e, k) fresh and used 1Pt-MoO_3-Mo_2N catalysts; (f, l) fresh and used 2Pt-MoO_3/Mo_2N catalysts.

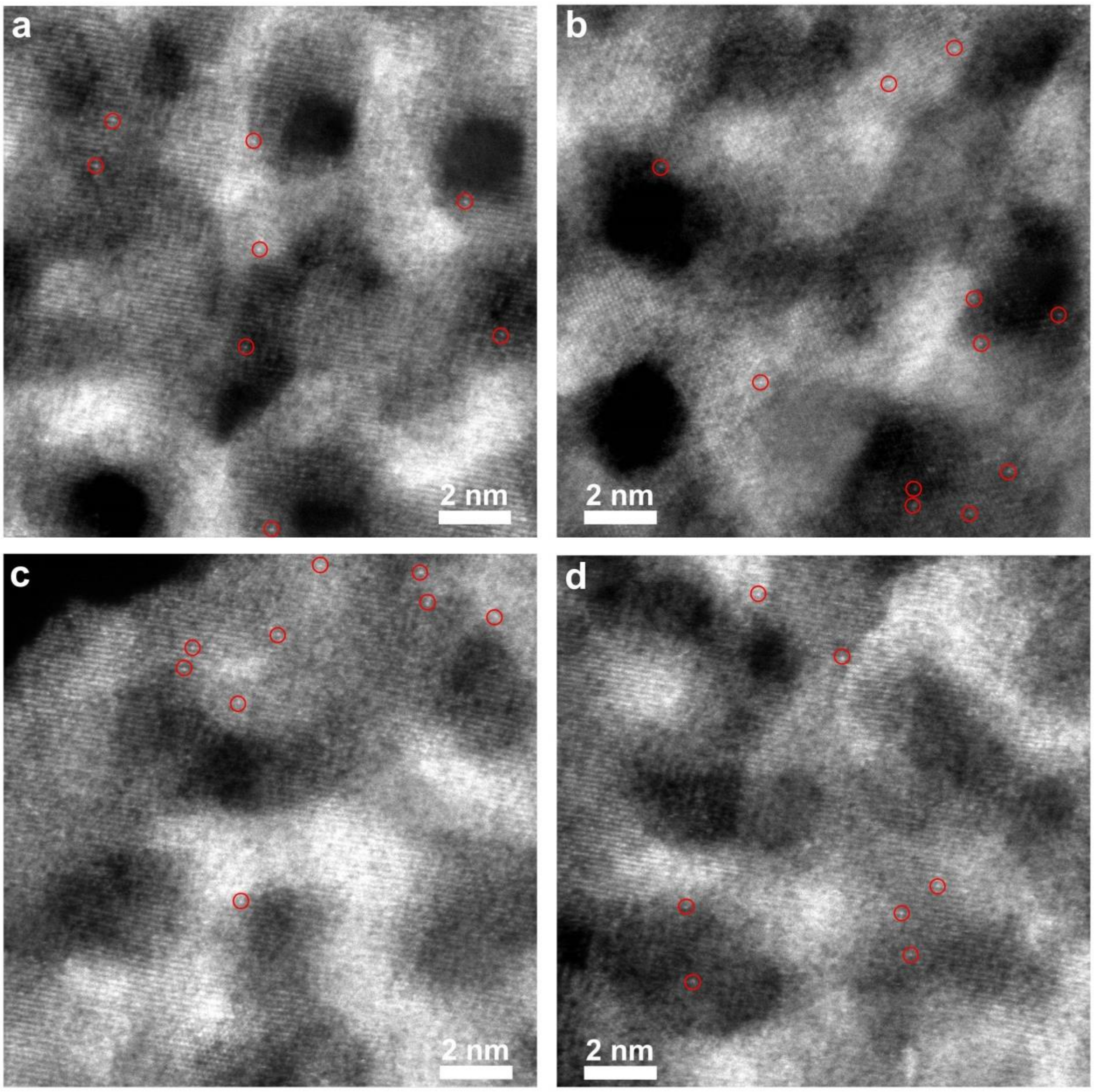

Supplementary Figure 2. HAADF-STEM images of the fresh 0.5Pt-MoO_3/Mo_2N catalyst. (a–d) 0.5Pt-MoO_3/Mo_2N before the RWGS reaction.

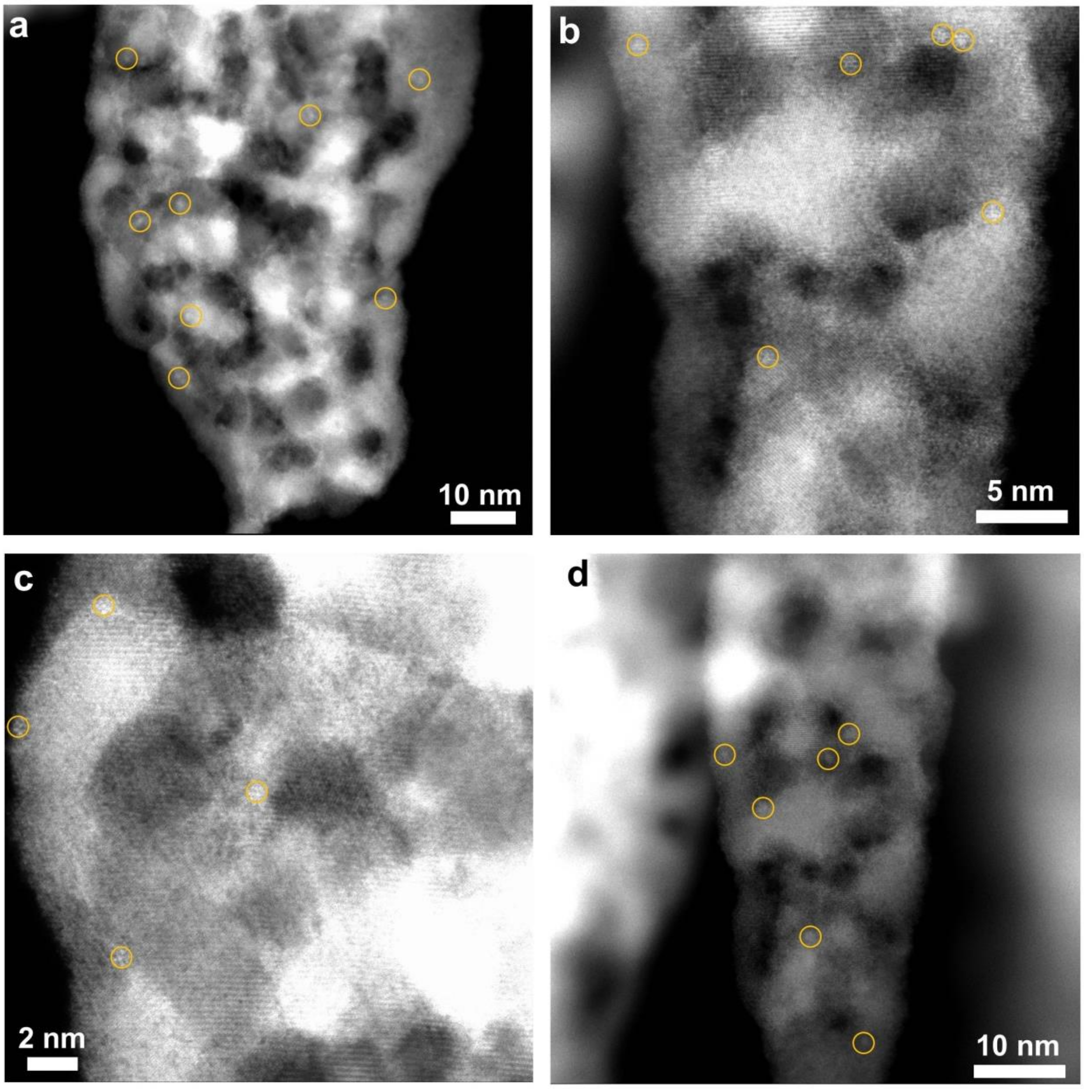

Supplementary Figure 3. HAADF-STEM images of the used 0.5Pt-MoO_3/Mo_2N catalyst. (a–d) 0.5Pt-MoO_3/Mo_2N after the RWGS reaction.

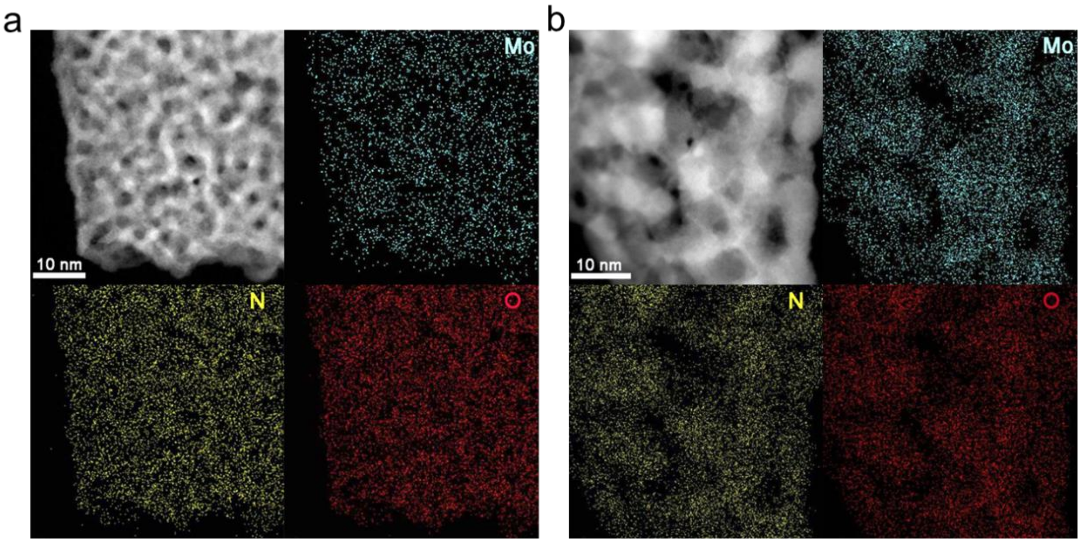

Supplementary Figure 4. STEM-EDS elemental mapping images of 0.5Pt-MoO_3/Mo_2N. (a) before the RWGS reaction and (b) after the RWGS reaction.

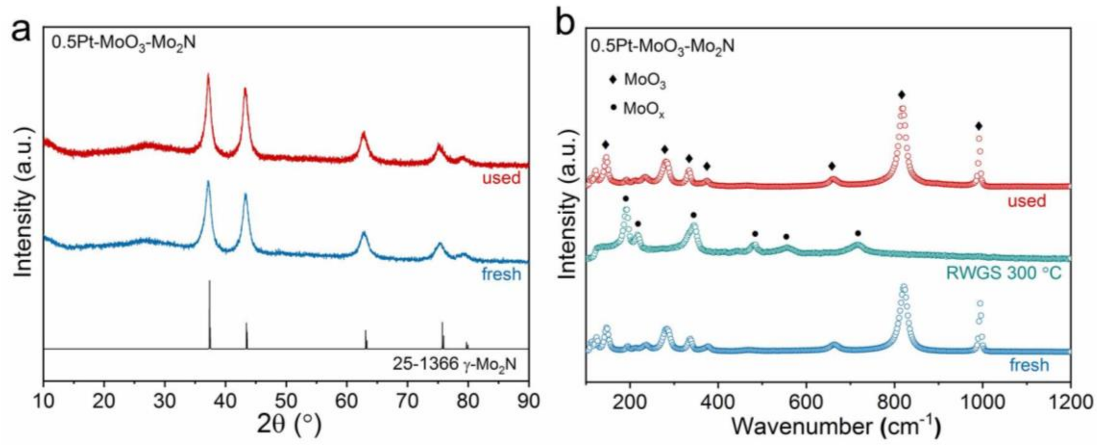

Supplementary Figure 5. The bulk phase and surface structure of 0.5Pt-MoO_3/Mo_2N. (a) XRD results of fresh and used 0.5Pt-MoO_3/Mo_2N catalysts; (b) *ex situ* and *in situ* Raman results of the 0.5Pt-MoO_3/Mo_2N catalysts.

XRD results showed that the bulk structure of the catalyst was Mo_2N, but the Raman results indicated that the surface structure of the catalyst was MoO_3, which proved the existence of a thin MoO_3 structure on the surface of Mo_2N. Meanwhile, during the RWGS reaction, the Raman signal changed from MoO_3 to MoO_x, which suggested that the surface MoO_3 lost oxygen and transformed to MoO_x structure during the reaction.

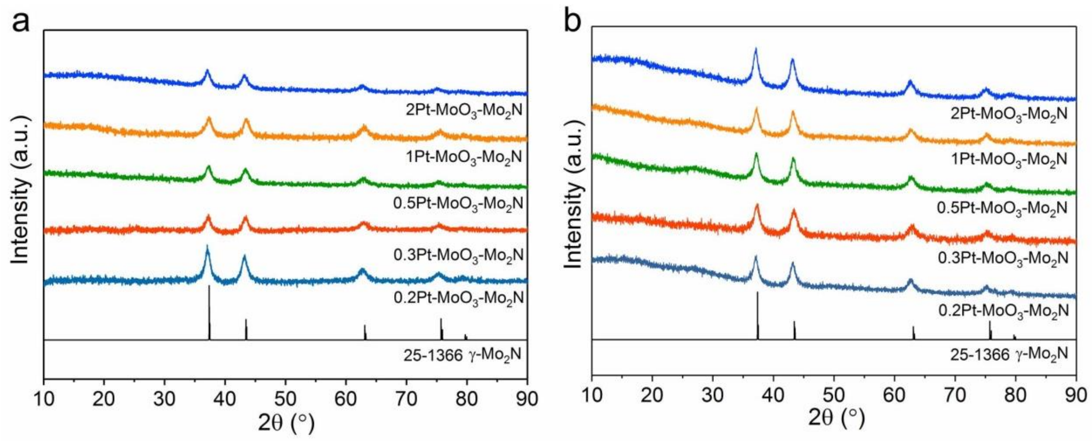

Supplementary Figure 6. The XRD patterns of Pt-MoO_3/Mo_2N catalysts. (a) before the RWGS reaction and (b) after the RWGS reaction.

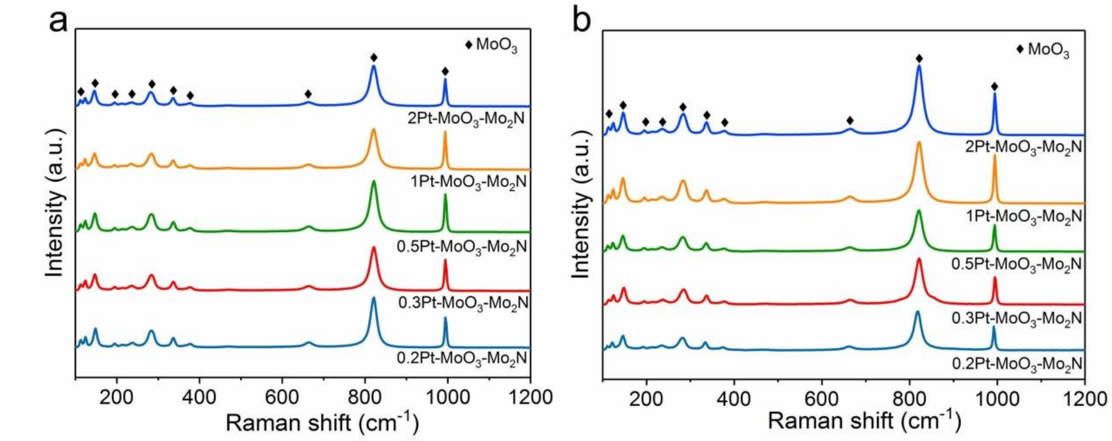

Supplementary Figure 7. Raman results of Pt-MoO_3/Mo_2N catalysts. (a) before the RWGS reaction and (b) after the RWGS reaction.

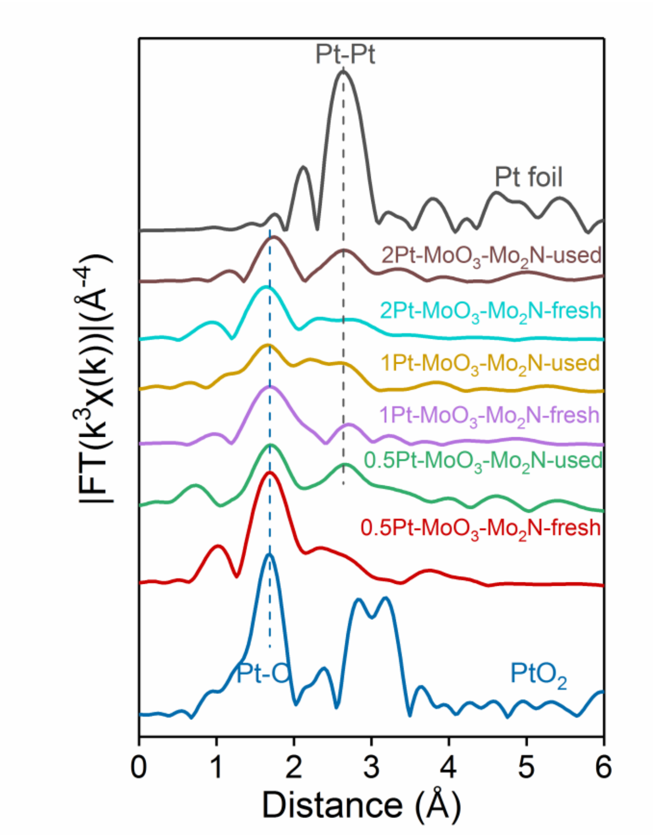

Supplementary Figure 8. The coordination structure of different samples. EXAFS spectra in *R* space for Pt L_3-edge of the fresh and used Pt-MoO_3-Mo_2N catalysts. Pt foil and PtO_2 are used as references.

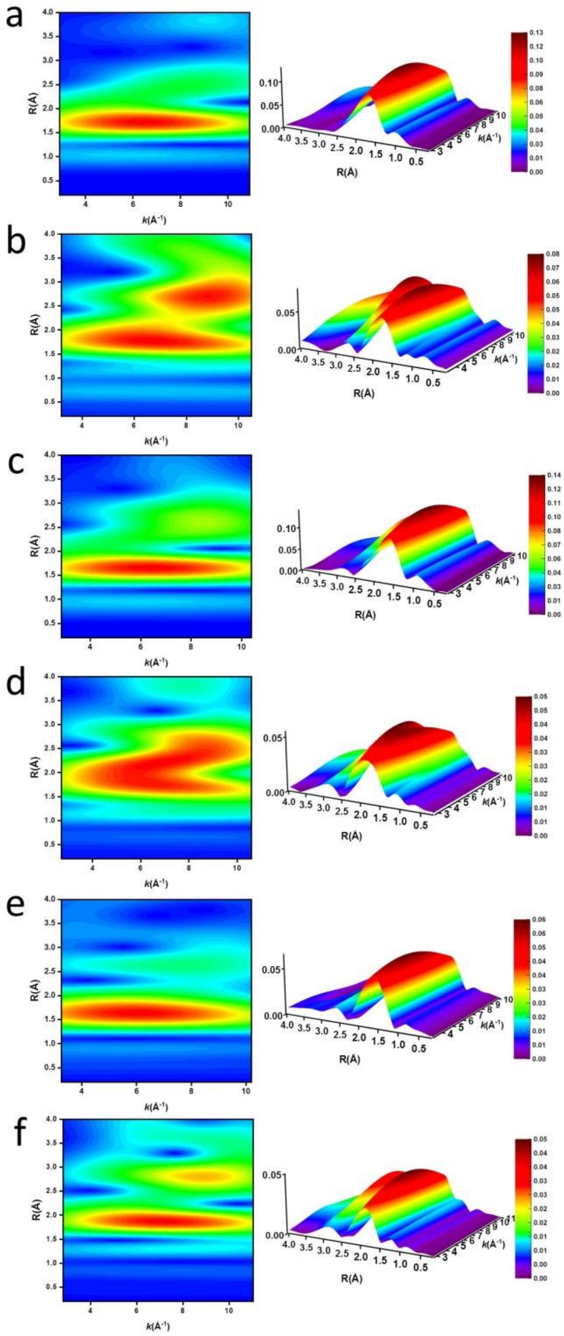

Supplementary Figure 9. Wavelet transformation (WT) EXAFS oscillation of Pt L3 edge in Pt-MoO_3/Mo_2N catalysts[10]. (a) fresh 0.5Pt-MoO_3/Mo_2N; (b) used 0.5Pt-MoO_3/Mo_2N after the RWGS reaction; (c) fresh 1Pt-MoO_3/Mo_2N; (d) used 1Pt-MoO_3/Mo_2N after the RWGS reaction; (e) fresh 2Pt-MoO_3/Mo_2N; (f) used 2Pt-MoO_3/Mo_2N after the RWGS reaction.

Wavelet transformation (WT) EXAFS oscillations of Pt L3 edge exhibited an increase in the intensity of Pt-Pt peak of the catalysts after the RWGS reaction compared to the catalyst before the reaction, which further indicated the aggregation of Pt atoms.

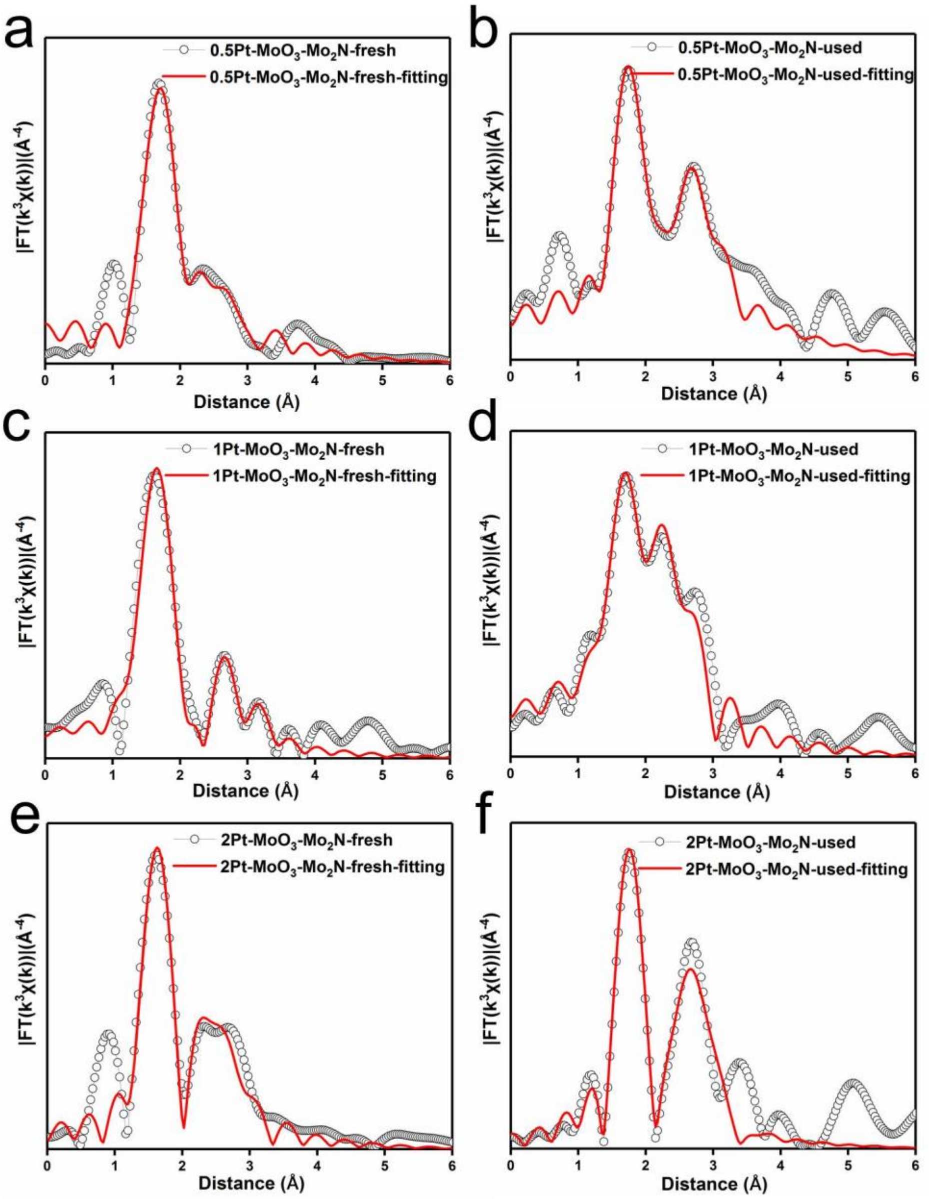

Supplementary Figure 10. EXAFS fitting results of Pt L_3 edge in Pt-MoO_3/Mo_2N catalysts. (a) fresh 0.5Pt-MoO_3/Mo_2N; (b) used 0.5Pt-MoO_3/Mo_2N after the RWGS reaction; (c) fresh 1Pt-MoO_3/Mo_2N; (d) used 1Pt-MoO_3/Mo_2N after the RWGS reaction; (e) fresh 2Pt-MoO_3/Mo_2N; (f) used 2Pt-MoO_3/Mo_2N after the RWGS reaction.

For all Pt-MoO_3/Mo_2N catalysts with different Pt loading, the stronger intensity of the Pt-Pt coordination peak of the used catalysts than that of the fresh catalysts was confirmed, suggesting the reduction and aggregation of the Pt species in the RWGS reaction.

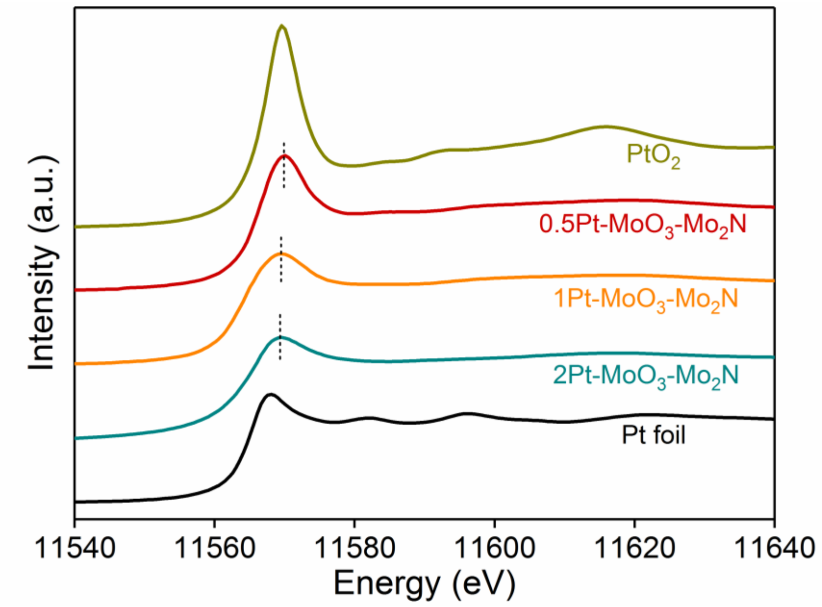

Supplementary Figure 11. The chemical state of Pt species in different samples. Pt L_3-edge XANES spectra for the fresh Pt-MoO_3/Mo_2N catalysts. Pt foil and PtO_2 are used as references.

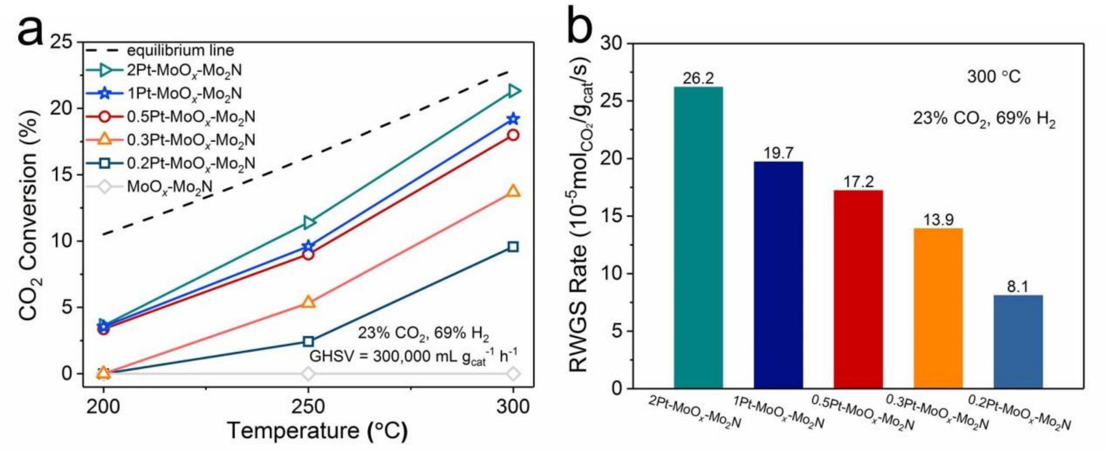

Supplementary Figure 12. Catalytic performance of various catalysts. (a) CO_2 conversion over different catalysts at various temperatures. (b) The mass specific activities of all Pt-MoO_x/Mo_2N catalysts.

The activity of the catalyst increased with the increase of Pt loading. However, when the Pt loading exceeded 0.5%, the catalyst activity became less influenced by the Pt loading, which might be caused by the aggregation of Pt species.

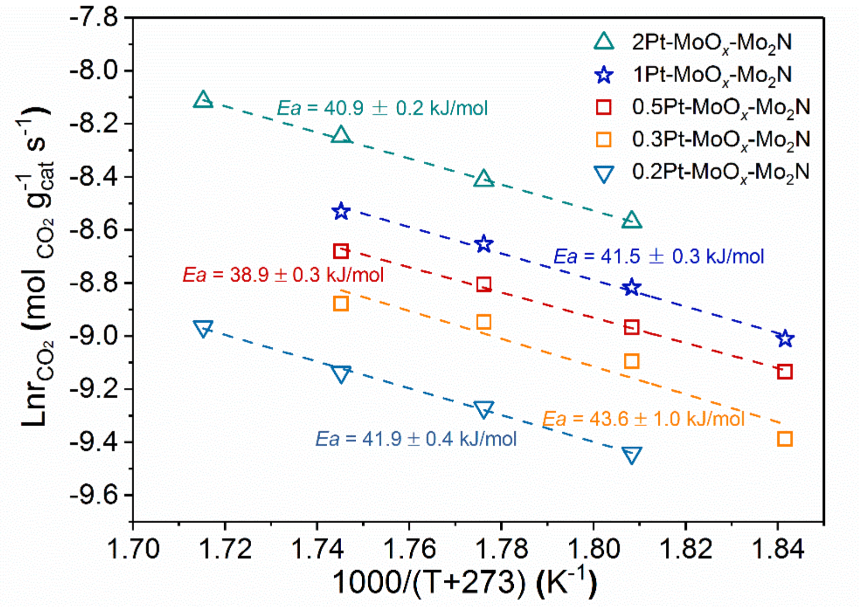

Supplementary Figure 13. Comparison of apparent activation energy (*E*a) over different catalysts. The apparent activation energy (*E*a) of catalysts with different Pt content.

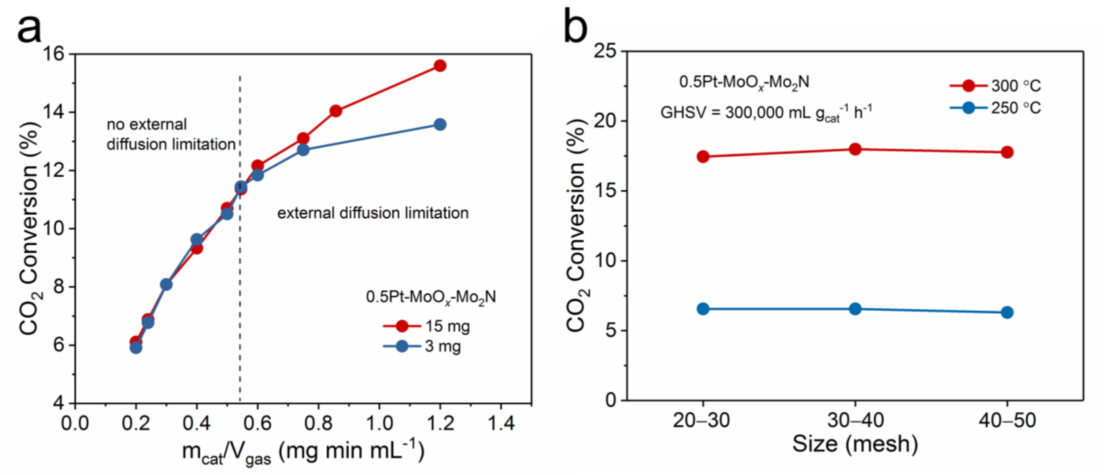

Supplementary Figure 14. Experiments for the absence of mass transfer limitation over the 0.5Pt-MoO$_x$/Mo$_2$N catalyst. (a) flow rate tests. (b) particles size tests.

In this work, the particle size of the catalysts used in the catalytic tests was about 20—40 mesh. In the particle size tests, the catalysts with different particle sizes showed similar catalytic activity, indicating that the effect of mass transfer limitation in the kinetic test has been excluded (Supplementary Figure 14a). Besides, in the flow rate tests, when the gas hourly space velocity (GHSV) was above 110,000 mLg_{cat}^{-1} h^{-1}, the effect of external diffusion could be excluded (Supplementary Figure 14b).

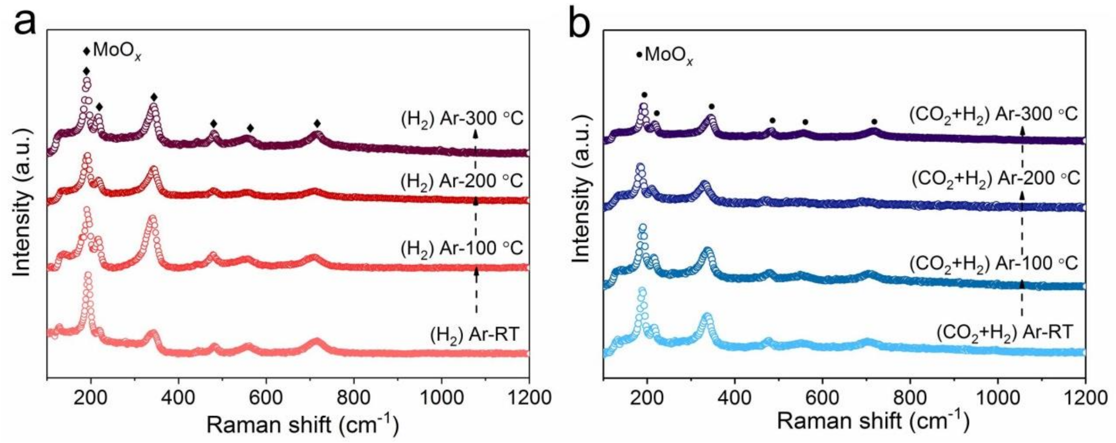

Supplementary Figure 15. *In situ* Raman results of 0.5Pt-MoO$_x$/Mo$_2$N. (a) under 5% H_2/Ar and (b) under the RWGS atmosphere.

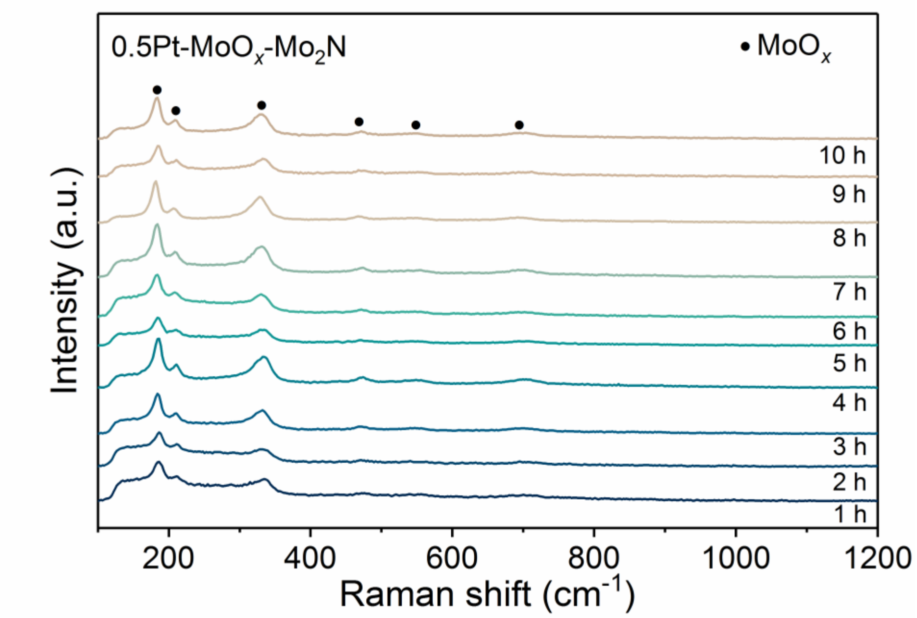

Supplementary Figure 16. The surface structure of 0.5Pt-MoO$_x$/Mo$_2$N under the RWGS reaction. *In situ* Raman results of 0.5Pt-MoO$_x$/Mo$_2$N under the RWGS atmosphere for 10 h.

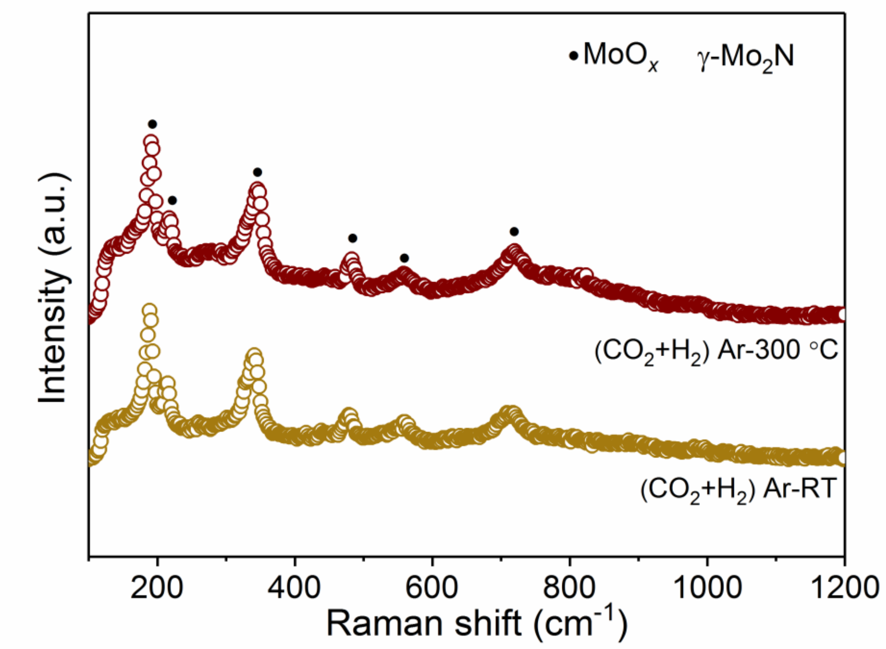

Supplementary Figure 17. The surface structure of *γ*-Mo_2N under the RWGS reaction. *In situ* Raman tests of the pure *γ*-Mo_2N support under the RWGS reaction.

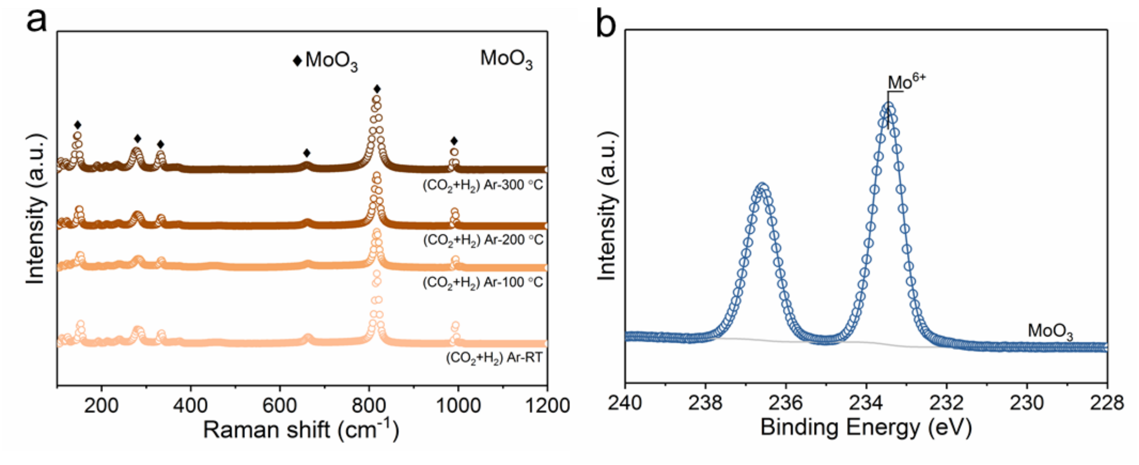

Supplementary Figure 18. The characterization of MoO_3. (a) *In situ* Raman result of MoO_3 under the RWGS reaction atmosphere; (b) Mo 3d XPS result of the MoO_3 sample.

When the MoO_3 sample was treated with RWGS reaction gas, the signal of MoO_3 did not disappear and no signal of MoO_x was generated, which indicated that the surface of MoO_3 was difficult to be reduced during the RWGS reaction, and thus no oxygen vacancy could be generated (Supplementary Figure 18a). Meanwhile, for the MoO_3 sample, the Mo 3d XPS spectra only exhibited the existence of Mo^{6+} (Supplementary Figure 18b), indicating that there was no oxygen vacancy on the surface of MoO_3.

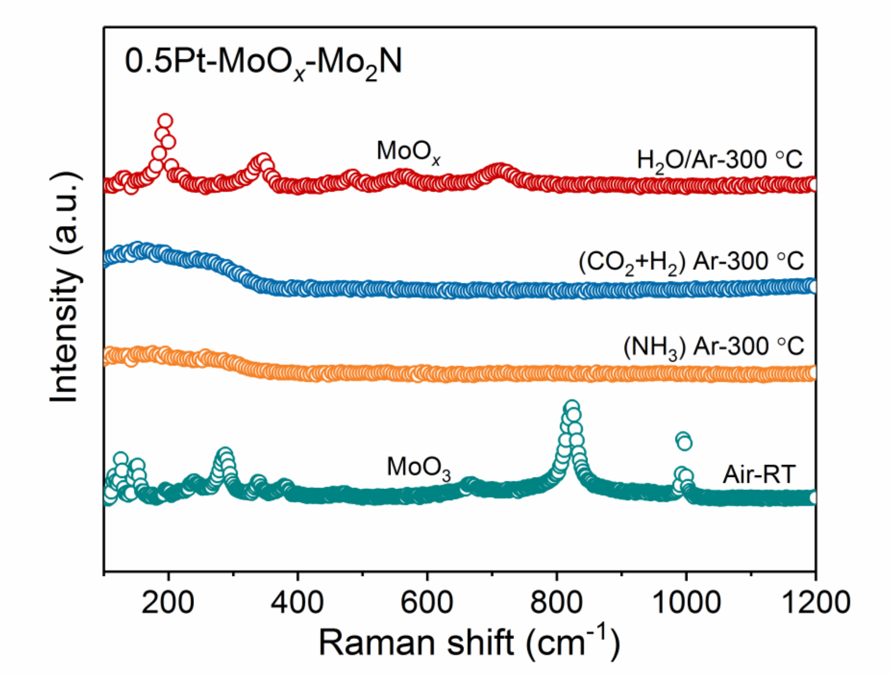

Supplementary Figure 19. The evolution of surface structure of 0.5Pt-MoO$_x$/Mo$_2$N under various atmospheres. *In situ* Raman tests of 0.5Pt-MoO$_x$/Mo$_2$N under various atmospheres.

As shown in Supplementary Figure 19, the Raman characteristic peaks of the MoO_3 disappeared when the 0.5Pt-MoO$_x$/Mo$_2$N catalyst was treated by NH_3, indicating the nitration for the MoO$_x$. When the nitrided surface was flowingly treated by ~3% H_2O/Ar, the MoO$_x$ peaks were regenerated again, reflecting the support surface was reoxidized by the low concentration of water vapor.

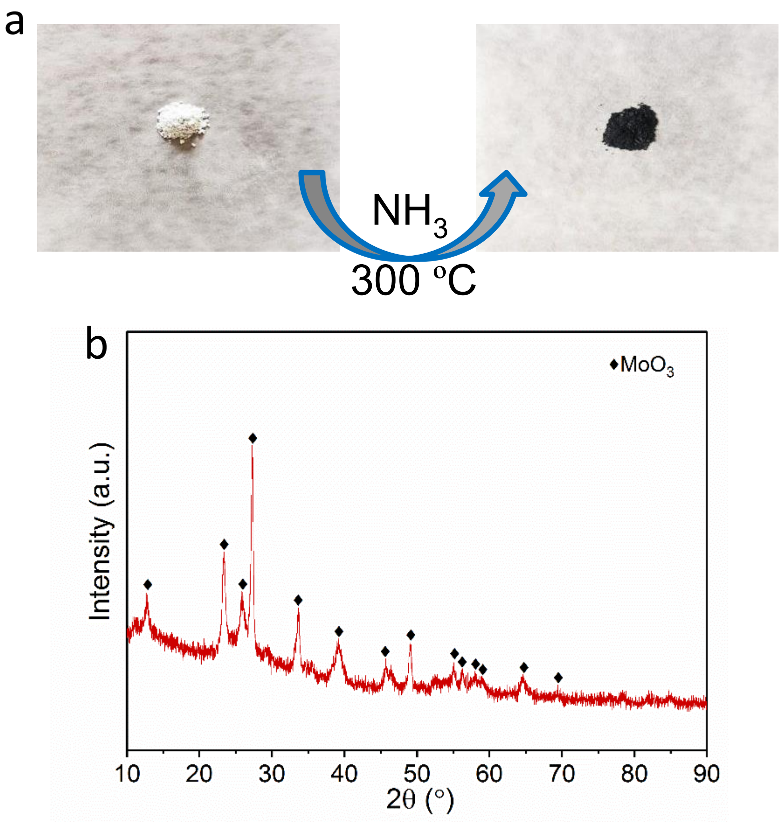

Supplementary Figure 20. The structural change of MoO_3 after the NH_3 treatment. (a) Color change of the MoO_3 sample treated with NH_3 flow at 300 ºC for 30 min. (b) The XRD result of the MoO_3 sample treated with NH_3 flow at 300 ºC for 30 min.

After the MoO_3 sample was treated with NH_3 at 300 ºC for 30 min, the color of MoO_3 changed from white to black, indicating that the surface of MoO_3 was nitrated by NH_3 (Supplementary Figure 20a). However, the XRD results showed that the bulk phase structure of the sample was still MoO_3 after the treatment by NH_3, which indicated that the NH_3 could only nitride the surface of MoO_3 at 300 ºC rather than the bulk structure (Supplementary Figure 20b).

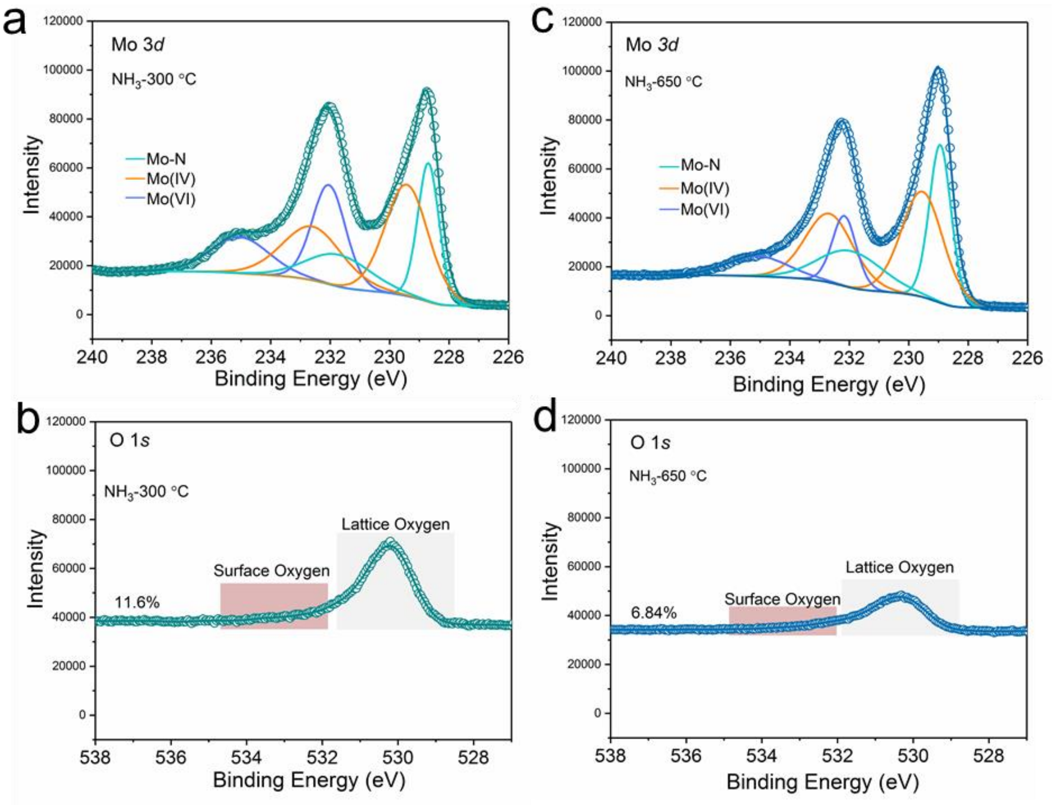

Supplementary Figure 21. Quasi *in situ* XPS spectra of 0.5Pt-MoO_x/Mo_2N. (a, b) Mo 3d and O 1s spectra after treatment under NH_3 at 300 ºC for 10 h; (c, d) Mo 3d and O 1s spectra after treatment under NH_3 at 650 ºC for 1 h.

After the nitration of ammonia at 300 ºC, the content of oxygen atoms on the catalyst surface decreased, demonstrating the occurrence of nitration (Supplementary Figure 21b). However, after the nitration of ammonia at 650 ºC for 1 h, the presence of oxygen atoms on the surface indicated that oxygen atoms could not be completely nitrided by NH_3 treatment at 650 ºC for 1 h (Supplementary Figure 21d).

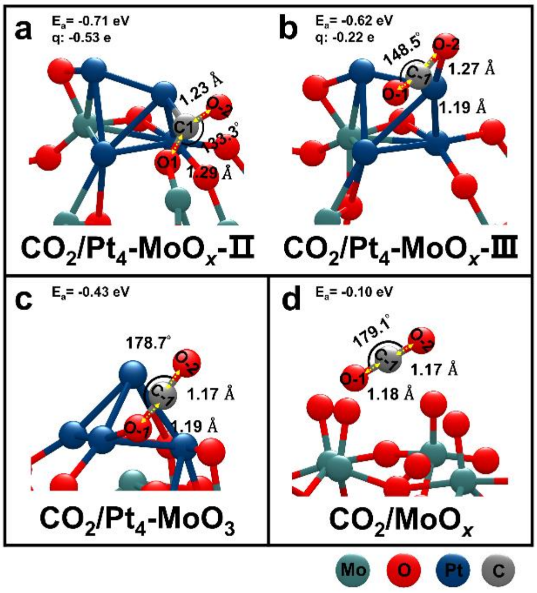

Supplementary Figure 22. Adsorption structures of a CO_2 molecule on different surfaces. (a, b) Pt_4-MoO_x surface; (c) Pt_4-MoO_3 surface; (d) MoO_x surface. E_a: adsorption energy of CO_2; q: calculated number of charges carried by CO_2 via the Bader charge analysis.

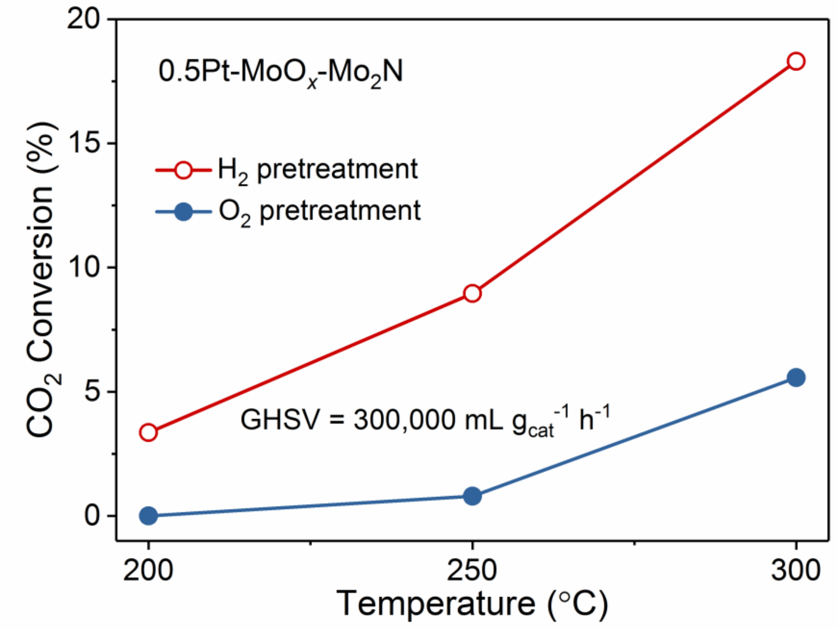

Supplementary Figure 23. The effect of pretreatment atmospheres on catalytic activity. The catalytic performance of 0.5Pt-MoO_x/Mo_2N after the pretreatment with 5% H_2/Ar and 1% O_2/Ar, respectively.

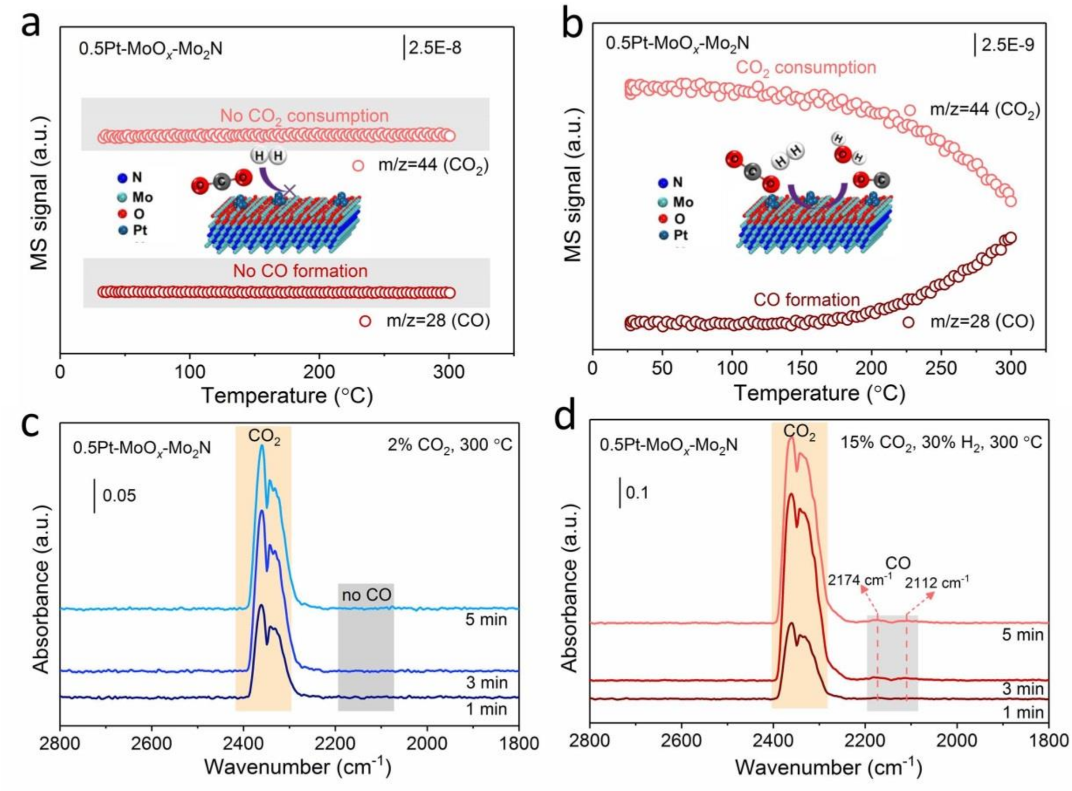

Supplementary Figure 24. Experiments to determine the mechanism of the RWGS reaction catalyzed by 0.5Pt-MoO_x/Mo_2N. (a) The CO_2 dissociation experiment of 0.5Pt-MoO_x/Mo_2N; (b) Temperature programmed surface reaction (TPSR) result of 0.5Pt-MoO_x/Mo_2N; (c, d) *In situ* diffused reflectance infrared Fourier transform spectroscopy (DRIFTS) spectra of 0.5Pt-MoO_x/Mo_2N during CO_2 treatment and reaction conditions at 300 °C, respectively.

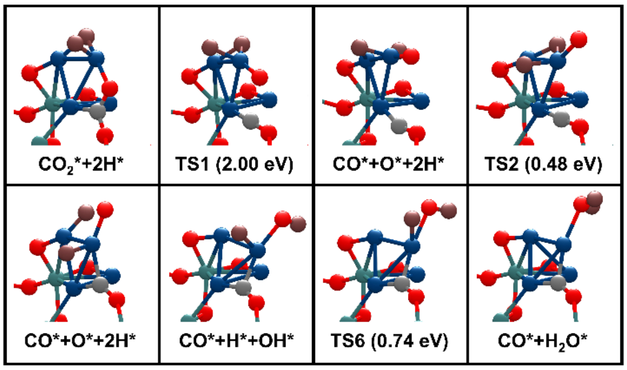

Supplementary Figure 25. Geometries of intermediates and transition states involved in the redox reaction pathway. The energy values in the parentheses refer to the energy barriers in the corresponding elementary steps. The Pt, Mo, C, O, and H atoms are depicted in blue, teal, gray, red, and brown, respectively.

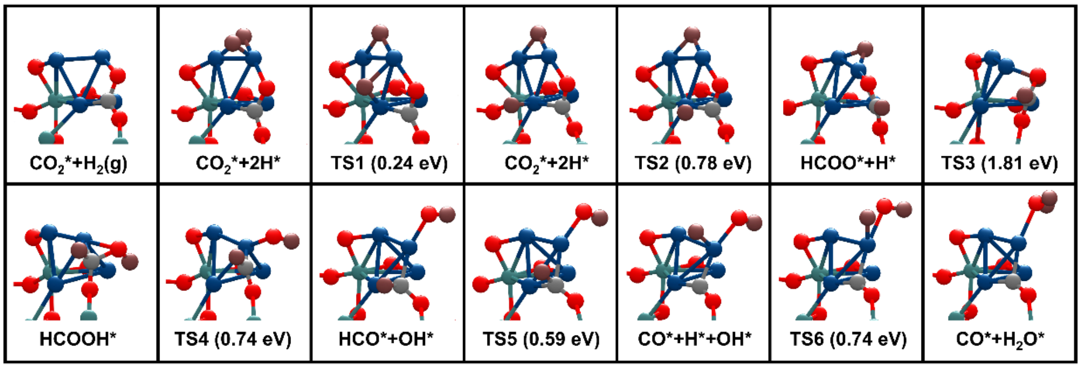

Supplementary Figure 26. Geometries of intermediates and transition states involved in the formate reaction pathway. The energy values in the parentheses refer to the energy barriers in the corresponding elementary steps. The Pt, Mo, C, O, and H atoms are depicted in blue, teal, gray, red, and brown, respectively.

Supplementary Tables:

Supplementary Table 1. Physical properties of the 0.5Pt-MoO_3/Mo2N catalyst.

Catalyst	Loading (wt.%)	BET surface area (m^2/g)
0.5Pt-MoO_3/Mo_2N	0.59[a]	78.1

[a]Determined by ICP-AES.

Supplementary Table 2. Structure parameters obtained from the EXAFS fitting.

Sample	Shell	Bond length (Å)	Coordination Number	σ^2 ($Å^2$)	E_0 shift (eV)	R-factor ($*10^{-3}$)
**0.5Pt-MoO_3/Mo_2N-fresh**	Pt-O	2.04	3.5	0.008	6.7	17.7
**0.5Pt-MoO_3/Mo_2N-used**	Pt-O	2.03	2.1	0.005	8.7	17.8
	Pt-Pt	2.84	1.6	0.007		
**1Pt-MoO_3/Mo_2N-fresh**	Pt-O	2.04	2.9	0.004	11.8	15.7
	Pt-Pt	2.83	2.1	0.005		
**1Pt-MoO_3/Mo_2N-used**	Pt-O	2.03	1.8	0.012	-5.8	18.2
	Pt-Pt	2.84	2.6	0.006		
**2Pt-MoO_3/Mo_2N-fresh**	Pt-O	2.02	2.2	0.008	6.9	17.9
	Pt-Pt	2.85	2.5	0.009		
**2Pt-MoO_3/Mo_2N- used**	Pt-O	2.03	1.2	0.002	9.3	21.9
	Pt-Pt	2.84	3.0	0.007		

Supplementary Table 3. Comparison of RWGS reaction for the as-prepared and literature reported catalysts.

Catalyst	H_2:CO_2	Temperature (ºC)	Pressure (MPa)	Rate (10^{-5}mol$_{CO}$/g$_{cat}$/s)
**0.5Pt-MoO_x/Mo_2N**	**3:1**	**250**	**0.1**	**7.7**
**0.5Pt-MoO_x/Mo_2N**	**3:1**	**300**	**0.1**	**17.2**
Cu/β–Mo_2C	2:1	300	0.1	7.3
Cu–Zn–Al	2:1	300	0.1	1.5
Pt/CeO_2	3:1	300	0.1	1.2
AuMo/SiO_2	2:1	300	0.8	0.6
In_2O_3-CeO_2	2:1	300	0.1	0.1
NiAu/SiO_2	3:1	340	0.1	0.8
Pt-CeO_2	4:1	400	0.1	4.8
TiO_2/Cu	3:1	400	0.1	1.8
SiO_2/Cu	3:1	400	0.1	1.1
Pt-TiO_2	1:1	400	0.1	0.5
Rh@S-1	3:1	450	1.0	0.3
Ni-in-Cu	3:1	500	0.1	4.0
NiAu/SiO_2	3:1	500	0.1	2.7
K_{80}–Pt/L	1:1	500	0.1	2.2
K_{200}–Pt/L	1:1	500	0.1	0.9
In_2O_3-CeO_2	2:1	500	0.1	0.3

Supplementary Table 4. Imaginary frequencies of all the transition state in the energy profiles shown in Figure 4f.

TS	TS1	TS2	TS3	TS4	TS5	TS6
Redox IF (cm^{-1})	318.8	29.6	--	--	--	1045.4
Carboxyl IF (cm^{-1})	953.6	260.6	867.7	--	--	--
Formate IF (cm^{-1})	413.8	236.1	660.0	190.8	521.2	1045.4

Supplementary References:

1. Kresse, G., Furthmuller, J. Efficiency of Ab-initio Total Energy Calculations for Metals and Semiconductors using a Plane-Wave Basis Set. *Comput. Mater. Sci.* **6**, 15−50 (1996).
2. Kresse, G.; Furthmüller, J. Efficient iterative schemes for ab initio total-energy calculations using a plane-wave basis set. *Phys. Rev. B* **54**, 11169–11186 (1996).
3. Blochl, P. E. Projector augmented-wave method. *Phys. Rev. B* **50**, 17953–17979 (1994).
4. Perdew, J. P., Burke, K., Ernzerhof, M. Generalized gradient approximation made simple. *Phys. Rev. Lett.* **77**, 3865–3868 (1996).
5. Monkhorst, H., Pack, J. Special points for Brillouin zone integrations. *Phys. Rev. B* **13**, 5188–5192 (1976).
6. Henkelman, G., Uberuaga, B. P. & Jónsson, H. A climbing image nudged elastic band method for finding saddle points and minimum energy paths. *J. Chem. Phys*. **113,** 9901–9904 (2000).
7. Henkelman, G., Jónsson, H. A dimer method for finding saddle points on high dimensional potential surfaces using only first derivatives. *J. Chem. Phys*. **111**, 7010–7022 (1999).
8. Momma, K. & Izumi, F. VESTA 3 for three-dimensional visualization of crystal, volumetric and morphology data. *J. Appl. Cryst*. **44**, 1272–1276 (2011).
9. Zhang, Z.-S., Fu, Q., Xu, K., Wang, W.-W., Fu, X.-P., Zheng X.-S., Wu, K., Ma, C., Si, R., Jia, C.-J., Sun, L.-D., Yan, C.-H. Intrinsically Active Surface in a Pt/γ-Mo_2N Catalyst for the Water–Gas Shift Reaction: Molybdenum Nitride or Molybdenum Oxide? *J. Am. Chem. Soc.* **142**, 13362–13371 (2020).
10. Funke, H., Scheinost, A. C. & Chukalina, M. Wavelet analysis of extended x-ray absorption fine structure data. *Phys. Rev. B* **71**, 094110 (2005).
